# Supplementary material for: Content-rich biological network constructed by mining PubMed abstracts
Source: BMC Bioinformatics. 2004 Oct 8;5:147. doi: 10.1186/1471-2105-5-147 (PMC528731; doi:10.1186/1471-2105-5-147)
Supplement: Additional File 2 — The original results of the above study (non-essential files are deleted to keep the file size under the limit set by BMC bioinformatics). [file 1471-2105-5-147-S2.bz2 › chilibotAdditionalFile2/dip50/280ID2498083E1103/html/FOSB_JUNB.html]

 


 **FOSB** and **JUNB** 
  
Found 123 abstracts in PubMed, retrieved 50.  
 

 What does Google say? 
 PDF only 
| .edu only 

---

**Interactive relationship** (e.g. stimulation, inhibition, etc)

**Neutral relationship**- Serum response factor is a transcription factor, which binds to a serum response element associated with a variety of genes including immediate early genes such as c fos,  **fosB** ,  **junB** , egr 1 and 2, neuronal genes such as nurr1 and nur77 and muscle genes such as actins and myosins.  Ref: 12120892 J Physiol Pharmacol, 2002
- Nuclear extracts isolated from hypoxic rat cardiac fibroblasts contained Fra1,  **JunB**  and also included  **FosB** .  Ref: 12371906 Biochem J, 2003

**Non-interactive relationship** (e.g. studied together, co-existance, homology, etc.)

- This study was carried out in order to examine the effects of acute or chronic L DOPA treatment on striatally expressed  **FosB**  and  **JunB**  like proteins in a rat model of Parkinson s disease.  Ref: 10579213 Neuroscience, 1999
- Phosphorylated c Jun,  **JunB** , JunD, and Fra 1, but not c Fos,  **FosB** , or ATF 2, are detected in the AP 1 DNA binding complex in cells exposed to trivalent arsenicals.  Ref: 12475910 FASEB J, 2003
- The DNA binding activities of c Fos,  **FosB**  Delta  **FosB** , Fra 1, Fra 2, and  **JunB**  were up regulated whereas JunD activity was decreased.  Ref: 11854297 J Biol Chem, 2002
- Immunoblot analyses confirmed that c Jun, JunD,  **JunB** , c Fos, Fra 1, Fra 2 and  **FosB**  immunoreactive proteins were present in whole cell extracts of all antral follicles and midluteal phase corpora lutea as well as granulosa cells isolated from different sized antral follicles.  Ref: 12606371 Biol Reprod< MedlineTA>Biol Reprod, 2002
- **FosB** , Fra 2, c Jun,  **JunB**  and, most abundantly, JunD bind the AP 1 EP in the absence and presence of serotonin.  Ref: 11162889 Mol Cell Endocrinol, 2000
- The increases of proENK and proDYN mRNA levels induced by KA were well correlated with the increases of c Fos, Fra 2,  **FosB** , c Jun, and  **JunB**  protein levels, which were significantly increased 3 h after KA administration and effectively inhibited by administration with melatonin.  Ref: 10902893 Hippocampus, 2000
- We assessed the role of NMDA receptors in the expression of NGFI A,  **junB**  and  **fosB**  mRNAs induced by light pulses of different intensities late in the night Zeitgeber Time ZT 18.  Ref: 10216223 Brain Res Mol Brain Res, 1999
- PACAP administration induced maximum expression of c fos,  **fosB**  and  **junB**  mRNA after 60 min, and of junD mRNA after 8 h.  Ref: 10657527 Neuropeptides, 1999
- Further analysis demonstrated that serum or 12 O tetradecanoylphorbol 13 acetate activation of several immediate early genes including fos,  **fosB** ,  **junB** , and egr1 was inhibited by Wnt signaling.  Ref: 11751871 J Biol Chem, 2002
- c Fos,  **FosB**  Delta  **FosB** , and  **JunB**  were associated with Smad4.  Ref: 11854297 J Biol Chem, 2002
- Expression of egr 1, egr 2, egr 3, c jun,  **junB** , c fos,  **fosB** , fra 1, fra 2, and CREB genes was analyzed by reverse transcription polymerase chain reaction in Schwann cells isolated from neonatal rat sciatic nerves and in the cell lines MSC 80, NIH 3T3, and CHO.  Ref: 11460264 Glia, 2001
- Double immunofluorescence labelling revealed that LiCl causes a coordinate upregulation of c Fos,  **FosB**  and  **JunB** , and these proteins are colocalized in a majority of cells examined in NTS, parabrachial nucleus PBN, and central nucleus of the amygdala CeA.  Ref: 10619667 Neuroreport, 1999
- c Fos, Fra 2,  **FosB**  and  **JunB**  compete for binding after photic stimulation.  Ref: 10869847 Neuroscience, 2000
- Similarly, in the second experiment it was found that the D1R dependent induction by AMPH of Fos,  **FosB** , and  **JunB** , but not NGFI A, in the NAc was enhanced in rats exposed 1 week earlier to repeated VTA AMPH.  Ref: 12112395 Synapse, 2002
- In the present study, the expression of members of the AP 1 family of transcription factors in breast tumors n = 53 was investigated by Western blot with antibodies specific for each of the AP 1 family members c jun,  **junB** , junD and c fos,  **fosB** , fra1 and fra2.  Ref: 10502734 Int J Cancer, 1999
- Supershift analysis of nuclear extracts revealed that bFGF stimulates the occupancy of AP 1 site by c Jun,  **JunB** , JunD, c Fos,  **FosB** , and Fra2.  Ref: 10830307 Endocrinology, 2000
- These studies demonstrate that a functional AP 1 site mediates MMP 2 transcription in cardiac cells through the binding of distinctive Fra1  **JunB**  and  **FosB**   **JunB**  heterodimers.  Ref: 12371906 Biochem J, 2003
- Anti  **FosB**  and  **JunB**  antibodies reacted weakly with the conjunctival epithelium.  Ref: 10435835 Curr Eye Res, 1999
- This effect was specific because palytoxin had little effect on c Jun,  **JunB** , JunD,  **FosB** , Fra 1, or Fra 2 binding or on overall levels of transcription factor binding.  Ref: 12460732 Toxicol Appl Pharmacol, 2002
- Administration of progesterone beginning on Day 20 prevented the onset of labor and blocked the expected rise in c fos,  **fosB** , fra 1, fra 2, and  **junB**  expression on Day 23.  Ref: 12080023 Biol Reprod, 2002
- Supershift experiments and Western blots confirmed that the binding of c Fos,  **JunB** , and JunD, but not of  **FosB** , was reduced in anergized cells.  Ref: 11685114 Transplantation, 2001
- Western analysis of the proteins in the nuclear extracts showed that the levels of c Jun,  **JunB** , JunD,  **FosB** , and Fra2 increased and the levels of c Fos and Fra1 decreased slightly with calcium treatment.  Ref: 10816578 J Biol Chem, 2000
- We also observed increased expression of mRNAs for Fos and Jun transcription factor family members c Fos,  **FosB** , Fra 2, and  **JunB** , as well as Fos family proteins in the entorhinal cortex after MK 801 administration.  Ref: 10911868 Ann N Y Acad Sci, 2000
- Normal lens epithelial cells expressed mRNA signals for junD, but not for c fos,  **fosB** , c jun, and  **junB** . mRNAs for c fos,  **fosB** , c jun, and  **junB**  were detected in the whole lens epithelium from the vicinity to the wound to the equator from 30 min to 8 hr post injury with their peaks after 30 min to 1 hr, but were no longer detected at 10 hr or later.  Ref: 11825018 Exp Eye Res, 2001
- Changes in the regional and compartmental distribution of  **FosB**  and  **JunB**  like immunoreactivity induced in the dopamine denervated rat striatum by acute or chronic L dopa treatment.  Ref: 10579213 Neuroscience, 1999
- To investigate whether both patterns of IEG expression are observed in the rat SCN, the authors studied the expression of NGFI A,  **junB** , c fos, and  **fosB**  near the time of subjective dawn in rats entrained to a light dark 12 12 cycle and then maintained in constant total darkness for approximately 48 h.  Ref: 10447307 J Biol Rhythms, 1999
- Gel shift and supershift analyses showed that the TRE binding factors and CRE binding factors comprised  **fosB** , c fos,  **junB** , and junD, and CRE binding protein and junD, respectively.  Ref: 10657527 Neuropeptides, 1999
- Using DNA protein gel shift assays we determined that nerve growth factor stimulates AP 1 binding in both PC12 and B5P cells, and identified c Fos,  **FosB** , Fra 1, Fra 2, c Jun,  **JunB**  and JunD in AP 1 complexes.  Ref: 11905996 J Neurochem, 2002
- Forty six globes were processed for in situ hybridization with oligonucleotide probes for c fos,  **fosB** , c jun,  **junB**  and junD mRNAs, and 60 globes were immunohistochemically analysed using anti c Fos and anti c Jun antibodies.  Ref: 11825018 Exp Eye Res, 2001
- The Wnt mediated repression of c Fos,  **FosB** , and  **JunB**  expression was consistent with a decrease in their binding to an AP 1 promoter element and decreased target gene transcription.  Ref: 11751871 J Biol Chem, 2002
- The labeling intensity of the c Jun, JunD, and Fra 2 antibodies was stronger than that of  **JunB** ,  **FosB** , and Fra 1.  Ref: 11036094 J Histochem Cytochem, 2000
- Supershift analysis of AP 1 DNA complexes with antibodies against the AP 1 sub units, c Fos,  **FosB** , Fra 1, Fra 2, c Jun,  **JunB** , and JunD, revealed that the AP 1 DNA complexes in the various clones had different compositions.  Ref: 10188725 Int J Cancer, 1999
- The present results show that L DOPA administration produces a long lasting increase in the levels of  **FosB** , but not  **JunB**  like immunoreactivity in the dopamine denervated striatum.  Ref: 10579213 Neuroscience, 1999
- PACAP potently stimulated the expression of c fos,  **fosB**   **junB**  and junD, but not c jun mRNAs, at doses of 0.1 10 nM, as revealed by Northern blot analysis.  Ref: 10657527 Neuropeptides, 1999
- Interaction between immunoglobulin like transcript 2 leukocyte Ig like receptor 1 on NKL cells and HLA B27 on target cells leads to an impairment of NKL natural cytotoxicity, which correlates with an absence of  **JunB** ,  **FosB** , and c Fos transcription, as well as an absence of their DNA binding activity.  Ref: 10201929 J Immunol, 1999
- We found that there is a lack of induction of c Fos,  **FosB** , Fra 2 and  **JunB**  by acute cocaine exposure, and of DeltaFosB by repeated cocaine administration in both the NAc and CPu of D1 receptor mutant mice compared with wild type control mice.  Ref: 12354293 J Neurochem, 2002
- However, NPY did not inhibit the induction of c fos,  **FosB**  or  **junB**  mRNA in any hippocampal region examined in the same animals, showing lack of correlation between immediate early gene induction and seizure activity.  Ref: 10471204 Neurosci Lett, 1999
- We examined by immunocytochemistry the localization of the AP 1 family proteins c Jun,  **JunB** , JunD, c Fos,  **FosB** , Fra 1, and Fra 2 in rat incisor ameloblasts.  Ref: 11036094 J Histochem Cytochem, 2000
- The expression of fos,  **fosB** ,  **junB** , and egr1 was also repressed in human colon tumors relative to patient matched normal tissue.  Ref: 11751871 J Biol Chem, 2002
- We have investigated the expression of the AP 1 transcription factor proteins, fos,  **fosB** , fra1, fra2, jun,  **junB** , junD, using Western blot analysis, in several types of asynchronously proliferating cells.  Ref: 11809534 Cancer Lett, 2002
- Expression of c fos,  **fosB** , fra 1, fra 2, and  **junB**  was low during early gestation, with a 5 to 10 fold increase on Day 23 during labor, and returned to low levels 1 day postpartum.  Ref: 12080023 Biol Reprod, 2002
- Fra 1, Fra 2,  **FosB** ,  **JunB** , JunD, c Jun, and c Fos levels are increased by EGCG treatment, as is AP1 factor binding to hINV promoter AP1 site.  Ref: 11698415 J Biol Chem, 2002
- Among the early response genes analyzed, c myc,  **junB** , junD, c jun, c fos,  **fosB** , fra, as well as max, mad1 4, sin3, only c jun and fra 2 mRNAs were up regulated after 5D exposure.  Ref: 12648525 J Steroid Biochem Mol Biol, 2003
- More importantly, these data show that striatal induction of  **FosB**  and  **JunB**  like proteins by chronic L DOPA treatment exhibits both regional and compartmental specificity.  Ref: 10579213 Neuroscience, 1999
- We further studied the expression of  **fosB** , c jun and  **junB** , in the AP 1 complex.  Ref: 12820376 Anticancer Res< MedlineTA>Anticancer Res, 1999
- Like  **FosB** ,  **JunB**  was induced in the DA denervated striatum by both acute and chronic L DOPA treatment, and exhibited similar distribution patterns.  Ref: 10579213 Neuroscience, 1999
- AP 1 stands as a synonym for different proteins such as c Jun,  **JunB** , JunD, c Fos,  **FosB**  as well as the Fos related antigens Fra 1 and Fra 2, which can either homo or heterodimerize to build up a functional transcription complex.  Ref: 10359524 Oncogene, 1999
- c Fos is a component of the dimeric transcription factor activator protein 1 Ap 1, which is composed mainly of Fos c Fos,  **FosB** , Fra 1 and Fra 2 and Jun proteins c Jun,  **JunB**  and JunD.  Ref: 10655067 Nat Genet, 2000
- MafG, ATF2,  **FosB** , and  **JunB**  were also detected in the arsenite complex.  Ref: 12220541 Arch Biochem Biophys, 2002
- After cell seeding on an extracellular matrix which allows polarization and expression of the mvdp gene in response to androgens, AP1 protein accumulation is greatly altered and consists in a loss of  **JunB** , Fra1,  **FosB**  and a decrease in c Fos, c Jun and Fra2, while JunD remained at the same level.  Ref: 10775801 J Steroid Biochem Mol Biol, 2000
- Using transcription analysis, such as multiplex messenger assays, we show here that natural cytotoxicity exerted by the human NKL cell line correlates with mRNA accumulation of very early activator protein 1 transcription factor genes such as  **JunB** ,  **FosB**  and c Fos.  Ref: 10201929 J Immunol, 1999
- The defect is secondary to an inability to phosphorylate CREB and to induce CREB dependent immediate early genes, including c jun,  **fosB** , fra2, and  **junB** , which are required for cytokine gene induction.  Ref: 11739719 Mol Cell Biol, 2002
- In unilaterally pregnant rats, stretch imposed by the growing fetus was found to increase the expression of c fos,  **fosB** , fra 1, fra 2, and  **junB**  only in the gravid horn on the day of labor.  Ref: 12080023 Biol Reprod, 2002
- Co transfection of cardiac fibroblasts with Fra1  **JunB**  and  **FosB**   **JunB**  expression plasmids led to significant increases in transcriptional activity.  Ref: 12371906 Biochem J, 2003
- RESULTS The normal corneal epithelium showed no staining with antibodies against c Fos, Fra 2,  **FosB** , c Jun or  **JunB** .  Ref: 10435835 Curr Eye Res, 1999
